# Analysis of Axillary Bud Germination Regulatory Network in Sugarcane Based on Transcriptome and Weighted Gene Co-Expression Network Analysis

**DOI:** 10.3390/plants15081200

**Published:** 2026-04-14

**Authors:** Yanye Li, Ting Yang, Zongtao Yang, Xujuan Li, Xin Lu, Jianming Wu, Jiayong Liu, Fenggang Zan, Yong Zhao, Jun Deng, Xinlong Liu

**Affiliations:** 1 Yunnan Key National Key Laboratory of Tropical Crop Breeding, Kunming 650205, China; liyanye2021@163.com (Y.L.);; 2 Laboratory of Sugarcane Genetic Improvement (Yunnan), Kaiyuan 661699, China; 3 Sugarcane Research Institute, Yunnan Academy of Agricultural Sciences, Kaiyuan 661699, China; 4 Sugarcane Research Institute, Guangxi Academy of Agricultural Sciences, Nanning 530007, China

**Keywords:** sugarcane, bud, transcriptome, hormone, tillering, transcription factor

## Abstract

Axillary bud germination in sugarcane is a critical agronomic trait that directly determines seedling emergence and tillering capacity; however, its molecular regulatory mechanisms remain poorly understood. In this study, we systematically investigated the hormonal dynamics and transcriptomic profiles of the sugarcane cultivar XTT22 across five developmental stages (from dormancy to the first new leaf stage). Our results revealed that abscisic acid (ABA) content fluctuated during germination, whereas indole-3-acetic acid (IAA) and gibberellin (GA) levels decreased significantly, suggesting their negative regulatory roles. In contrast, cytokinin (CTK) and ethylene (ETH) contents increased at the initiation stage, indicating positive promoting functions. Transcriptome analysis identified 31,513 differentially expressed genes (DEGs), which were significantly enriched in pathways related to hormone signal transduction, starch/sucrose metabolism, and photosynthesis. Weighted gene co-expression network analysis (WGCNA) constructed 12 co-expression modules, among which the antiquewhite4 module (negatively correlated with IAA, GA, and ABA contents) and the darkorange2 module (positively correlated with cytokinin content) were identified as key regulatory modules. From these modules, seven core hub transcription factors (e.g., *ScTCP5*, *ScSCR*, and *ScSHR1*) were screened, and their expression patterns were validated by RT-qPCR. Furthermore, the expression trends of six hormone-related DEGs were highly consistent with the RNA-seq data. Collectively, this study elucidates the hormonal dynamics and gene regulatory networks underlying axillary bud germination in sugarcane, providing candidate gene resources for breeding high-yield varieties with enhanced emergence and tillering capacity.

## 1. Introduction

Sugarcane (*Saccharum* spp.) is the world’s most important sugar crop, accounting for approximately 80% of global sugar production [1]. As a renewable bioenergy feedstock, its by-products also have broad application prospects in ethanol production, papermaking, and power generation [2]. Sugarcane is primarily propagated asexually, and the germination ability of axillary buds on stem nodes directly determines seedling emergence, perennial root number, and the final number of productive stalks, making it a core factor influencing yield [3,4]. Effective stalk number is determined by tillering dynamics, with early and frequent tillering being conducive to the formation of a high-yield population structure [4,5]. Therefore, elucidating the regulatory mechanism of axillary bud germination for high-yield breeding in sugarcane is essential.

In model plants and other cereals, the genetic framework governing axillary bud outgrowth has been extensively studied. For instance, genes such as TEOSINTE BRANCHED1 (*TB1*) in maize (*Zea mays* L.) and its rice (*Oryza sativa* L.) ortholog FINE CULM1 (*FC1*) act as key integrators of strigolactone signaling to repress tillering [6,7,8]. The MONOCULM1 (*MOC1*) gene in rice is essential for axillary bud formation [9,10], while the MORE AXILLARY GROWTH (MAX) pathway in Arabidopsis (*Arabidopsis thaliana* L.) defines the strigolactone biosynthesis and signaling cascade [11,12]. These studies have established that hormone crosstalk—particularly among auxin, cytokinin, and strigolactones—forms the core of the regulatory network controlling shoot branching. In crops, the extent of axillary bud outgrowth directly determines plant architecture and final yield components such as tiller or branch number [13,14]. In sugarcane, the germination of axillary buds is a critical agronomic trait that significantly impacts plant architecture, ratoon performance, and effective stalk numbers and cane yield [3]. Previous studies in sugarcane have indicated that hormones such as auxin and cytokinin are major signaling molecules affecting this process [15,16,17]. At the molecular level, the high expression of strigolactone pathway-related genes (e.g., *ScHTD2*, *ScF-box*, *ScD27*) has been found to inhibit axillary bud germination [18,19,20], while the *SoMADS57* gene may positively regulate tillering by suppressing this pathway [21]. These findings suggest that the core hormonal regulators identified in model plants are also relevant in sugarcane. However, the dynamic changes and synergistic mechanisms of multiple hormones—particularly gibberellin (GA), abscisic acid (ABA), and ethylene (ETH), whose roles in this process remain elusive—during the precise timing of axillary bud germination in sugarcane are still poorly understood. Therefore, a systematic investigation integrating hormone dynamics with gene expression profiles is urgently needed to elucidate the regulatory network underlying this key agronomic trait in this complex polyploid crop.

In recent years, high-throughput sequencing and bioinformatics have provided powerful tools for systematically deciphering the molecular mechanisms underlying complex traits. Transcriptome analysis combined with weighted gene co-expression network analysis (WGCNA) effectively identifies co-expressed gene modules and pinpoints core regulatory factors [22,23]. In this study, we integrated physiological (endogenous hormone) and transcriptomic data from five key developmental stages of sugarcane axillary buds to: (1) clarify the dynamic patterns of five key hormones (IAA, CTK, GA, ABA, ETH) during germination; (2) identify differentially expressed genes and their enriched biological pathways across developmental stages, revealing their association with hormone signaling; and (3) construct gene co-expression networks to screen for key hormone-regulated modules and pivotal transcription factors. These findings will lay a solid foundation for deepening our understanding of sugarcane axillary bud germination mechanisms and advancing molecular design breeding.

## 2. Results

### 2.1. Dynamic Changes in Endogenous Hormones During the Sprouting Process of Sugarcane Axillary Buds

**Figure 1:**
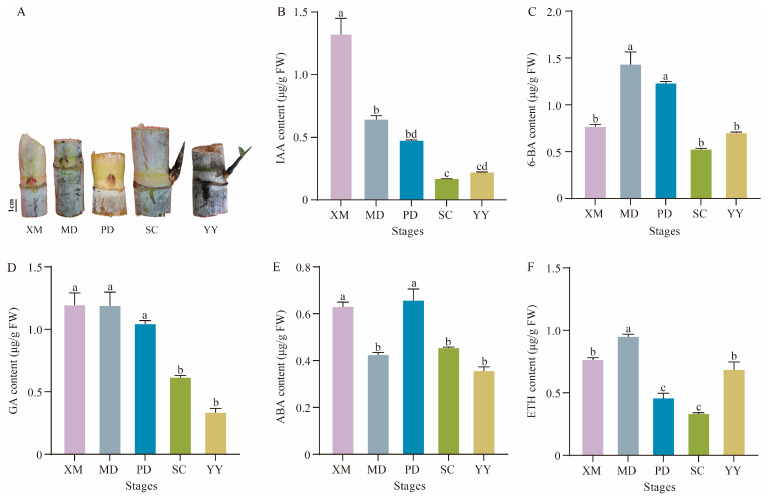
Phenotypic diagrams (**A**) and bar charts of endogenous hormones in sugarcane axillary buds at the XM, MD, PD, SC, and YY stages (**B**–**F**). XM: dormancy period; MD: stirring period; PD: swelled period; SC: elongation period; YY: the first new leaf period; error bars indicate standard errors. Statistical significance was determined by one-way ANOVA, and different letters above the bars indicate significant differences at *p* < 0.05.

Analysis of the content changes in five endogenous hormones across five developmental stages (Figure 1) revealed that IAA content peaked during dormancy at 1.30 μg/g FW. It declined to its lowest point during the elongation stage at 0.16 μg/g FW. Although it slightly rebounded during the new leaf stage, it remained significantly lower than during dormancy, indicating that low IAA levels promote germination. During the early stages of bud germination (MD and PD), 6-BA (CTK) content rapidly increased to a peak value of 1.44 μg/g FW. However, at the later stages (SC and YY), CTK levels were not significantly different from those at the dormancy stage (XM) (Figure 1C). These results suggest that CTK plays a key promoting role in the initiation of bud germination. GA content continuously decreased throughout the process, revealing its negative regulatory role. ABA content fluctuated, decreasing during the stirring stage, briefly rebounding during the swelled stage, and then declining again. ETH content increased transiently at the stirring stage (MD), decreased during the swelling and elongation stages (PD and SC), and then significantly rebounded during the first new leaf stage (YY) (Figure 1F).

### 2.2. Transcriptome Sequencing and Differential Gene Expression Analysis

Sequencing data from five samples exhibited high quality (Q30 > 92%, GC content > 55%), yielding a total of 121,103 unigenes (Table 1).

**Figure 2:**
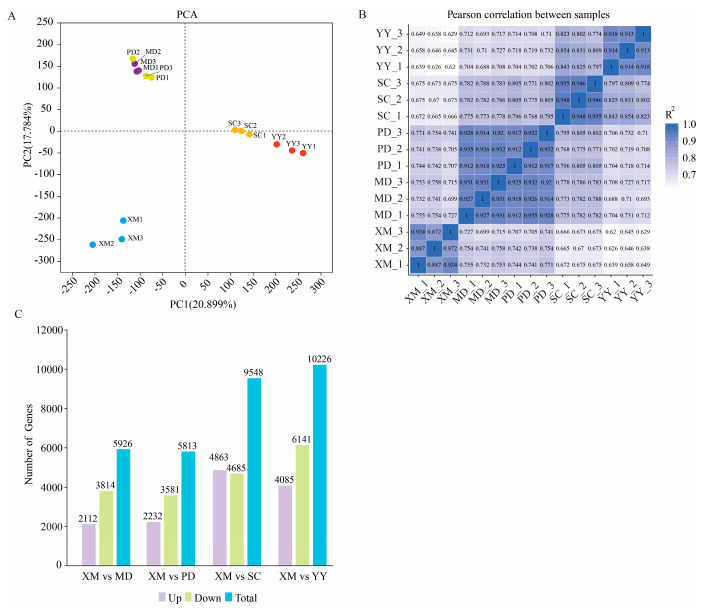
Transcriptome sequencing analysis. (**A**): PCA analysis; (**B**): Correlation analysis among samples; (**C**): Bar chart of DEGs between MD, PD, SC, and YY stages; XM: dormancy period, MD: stirring period, PD: swelled period, SC: elongation period, YY: the first new leaf period.

PCA analysis revealed that PC1 and PC2 explained 20.899% and 17.784% of the total variance, respectively. Materials from different groups were largely separated in the PCA plot: the dormancy stage (XM) was distinctly separated from other stages; the bud break stage (MD) clustered with the fruit enlargement stage (PD) but was not completely separated; the elongation stage (SC) and the emergence of the first new leaf stage (YY) clustered relatively close but were also clearly separated (Figure 2A). The correlation coefficients among samples were all greater than 0.8, indicating good reproducibility (Figure 2B). Using XM as the reference, 5926, 5813, 9548, and 10,226 DEGs were identified at the MD, PD, SC, and YY stages, respectively (Figure 2C). The increasing number of DEGs as development progressed, particularly during the SC and YY stages, suggests that the middle and late stages of germination involve more complex transcriptional reprogramming.

### 2.3. GO and KEGG Enrichment Analyses of DEGs

**Figure 3:**
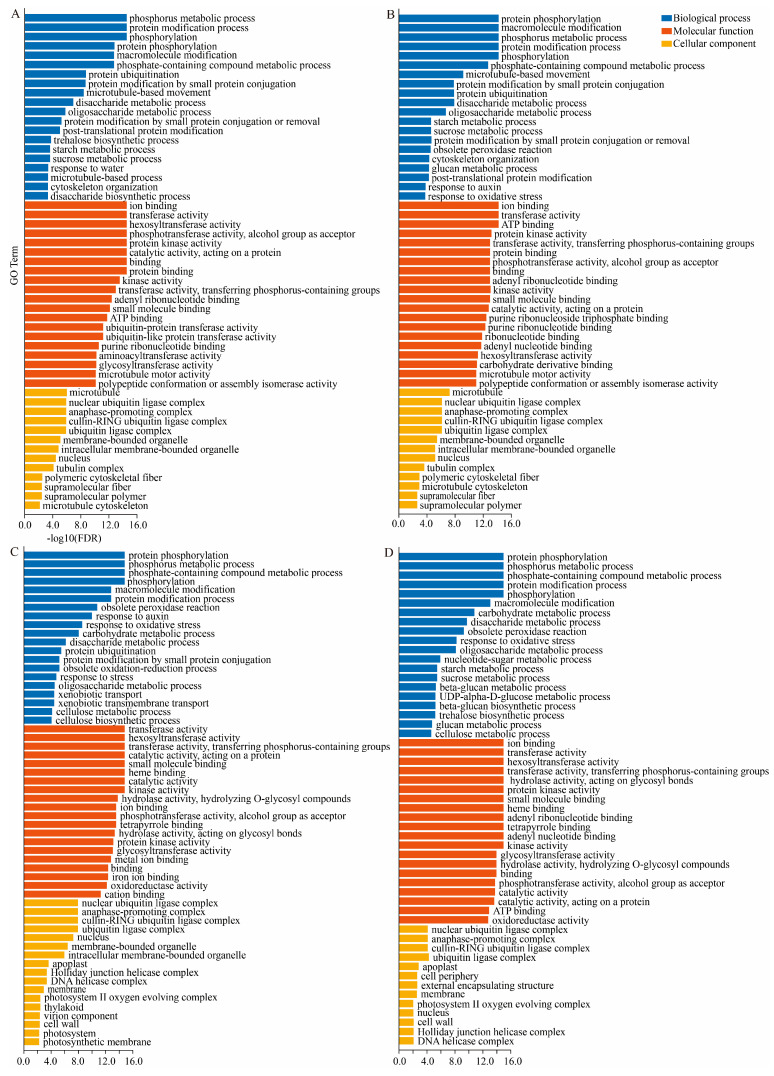
Top 20 GO pathways enriched in DEGs during axillary bud sprouting. The vertical axis represents enriched GO terms, while the horizontal axis represents the multiple testing-corrected *p*-value (FDR); (**A**): XM vs. MD; (**B**): XM vs. PD; (**C**): XM vs. SC; (**D**): XM vs. YY.

GO enrichment analysis revealed (Figure 3) that DEGs in the pre-germination stage (MD, PD) were significantly enriched in entries such as “protein phosphorylation (GO:0006468)”, “ubiquitination (GO:0016567)”, and “glycosyltransferase activity (GO:0016757)”, providing a foundation for cell activation and division, “glycosyltransferase activity (GO:0016757)”, and other entries, providing the foundation for cell activation and division. The late germination stage (SC, YY) additionally enriched entries such as “microtubule-based movement (GO:0007018)”, “cellulose metabolic process (GO:0030243)”, and “photosynthesis (GO:0015979)”, consistent with organ elongation and functional establishment.

**Figure 4:**
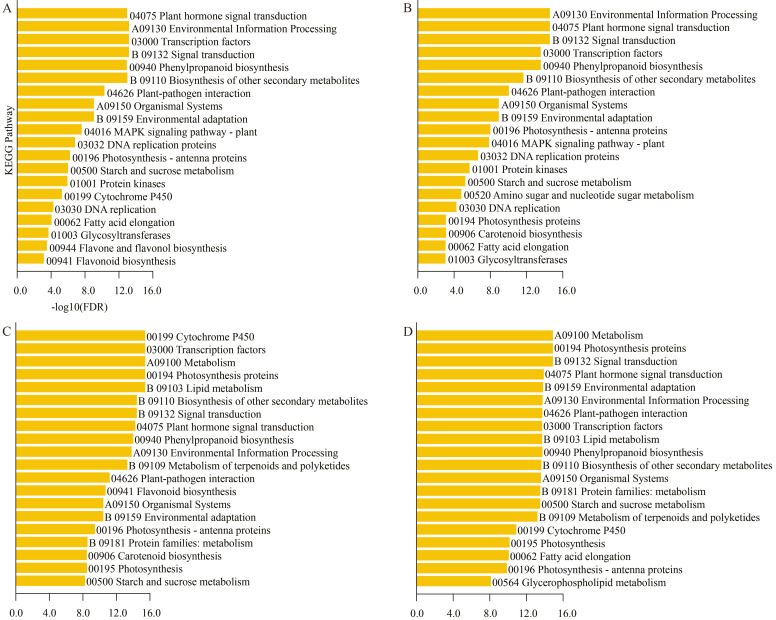
Top 20 KEGG pathways enriched in DEGs during sugarcane axillary bud sprouting. The vertical axis represents enriched KEGG pathways, while the horizontal axis represents the multiple testing-corrected *p*-value (FDR); (**A**): XM vs. MD; (**B**): XM vs. PD; (**C**): XM vs. SC; (**D**): XM vs. YY.

KEGG enrichment analysis revealed (Figure 4) that four comparison groups were commonly and significantly enriched in pathways such as plant hormone signal transduction (ko04075), starch and sucrose metabolism (ko00500), photosynthesis-antenna proteins (ko00196), and phenylpropanoid biosynthesis (ko00940). In addition, specifically significantly enriched pathways were identified at each stage, including flavone and flavonol biosynthesis (ko00944) at the MD stage, amino sugar and nucleotide sugar metabolism (ko00520) at the PD stage, and Glycerophospholipid metabolism (ko00564) at the YY stage, reflecting the metabolic characteristics of different developmental stages.

### 2.4. Differentially Expressed Genes in Hormone Signaling Pathways Regulating Axillary Bud Emergence in Sugarcane

**Figure 5:**
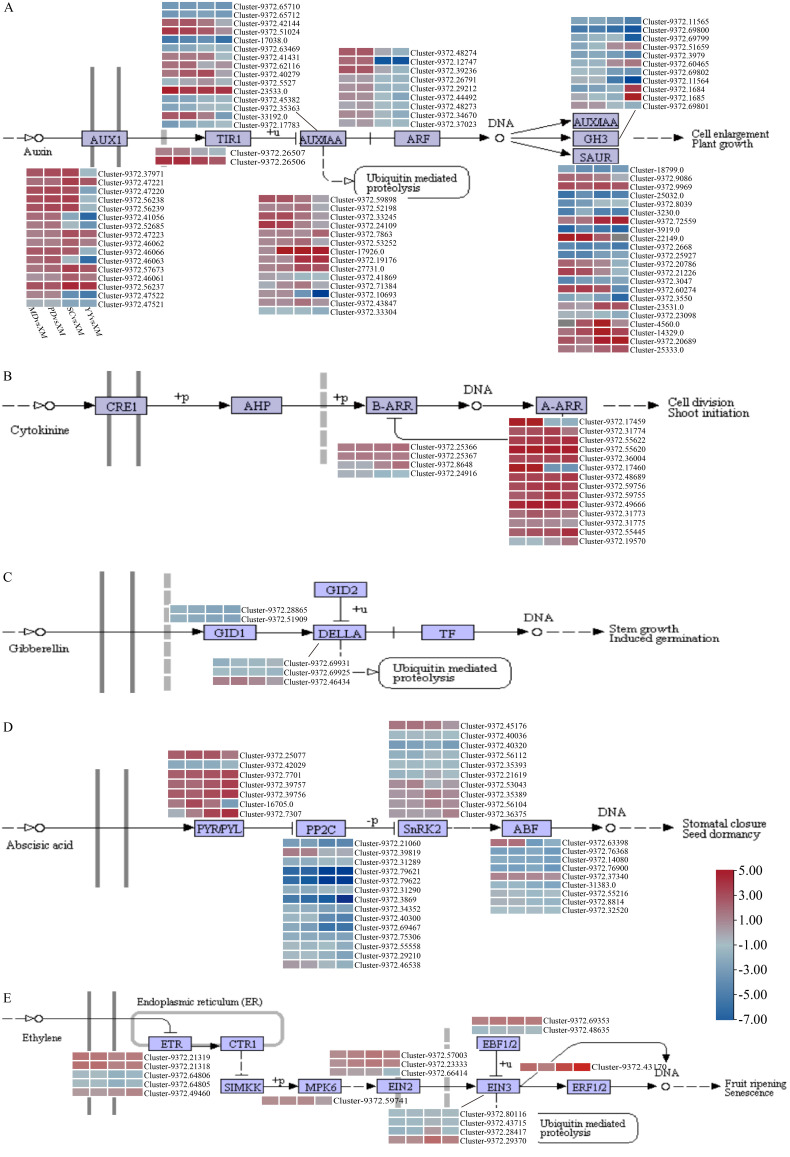
Temporal expression patterns of differentially expressed genes in hormone signaling pathways; Analysis of DEGs in IAA (**A**), CTK (**B**), GA (**C**), ABA (**D**) and ETH (**E**) signal transduction pathways; Red and blue blocks indicate upregulation and downregulation of relative expression levels; XM: dormancy period; MD: stirring period; PD: swelled period; SC: elongation period; YY: the first new leaf period.

Furthermore, we analyzed the plant hormone signaling pathways that showed significant enrichment in all four comparison groups of differentially expressed genes during sugarcane axillary bud emergence (Figure 5). The differentially expressed genes were primarily concentrated in the auxin (IAA), cytokinin (CTK), gibberellin (GA), abscisic acid (ABA), and ethylene (ETH) signaling pathways (Figure 5).

The IAA pathway contained 89 genes across the AUX1 (16 genes), TIR1 (2 genes), AUX/IAA (29 genes), ARF (9 genes), SAUR (22 genes), and GH3 (11 genes) families. Among these, most AUX1, TIR1, and AUX/IAA genes exhibited an expression pattern of “upregulation in the early stage and downregulation in the late stage” (Figure 5A). The CTK pathway contained 4 Type-B ARR genes and 14 Type-A ARR genes, with the majority (15/18) showing sustained upregulation throughout germination (Figure 5B). The GA pathway comprised 2 gibberellin receptor proteins (GID1) and 3 DELLA proteins, which exhibited sustained downregulation during germination (Figure 5C). The ABA pathway comprised 39 genes across the PYL (7 genes), PP2C (14 genes), SRK2 (10 genes), and ABF (9 genes) families. Most PYL family genes were upregulated, while both PP2C and ABF gene families were downregulated (Figure 5D). During ETH signal transduction, 17 genes were annotated to families including ETR (5 genes), MPK6 (1 gene), ERF1/2 (1 gene), EBF2 (2 genes), and EIN (7 genes), exhibiting differential expression patterns (Figure 5E).

### 2.5. Construction of Gene Co-Expression Networks and Analysis of Key Modules

**Figure 6:**
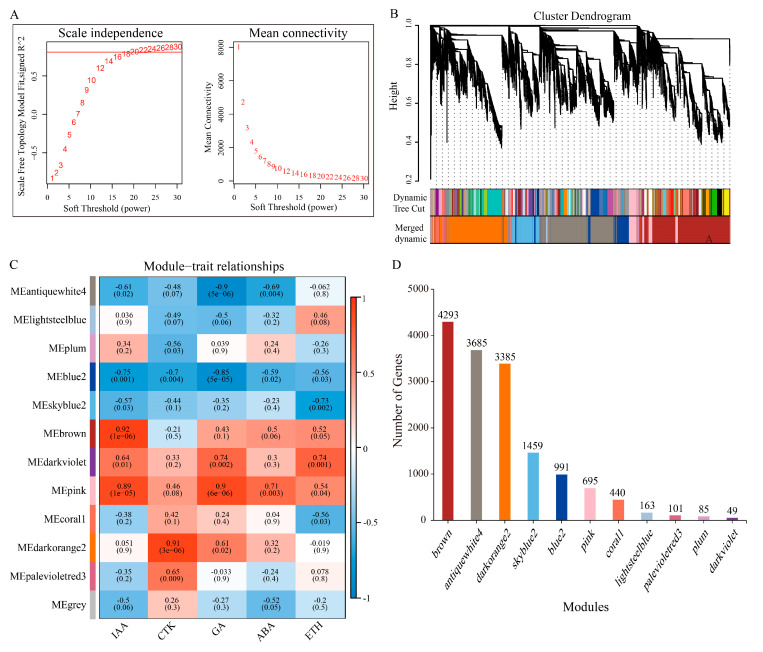
(**A**): Selection of the optimal soft threshold; (**B**): Systematic clustering tree and gene modules of differentially expressed genes, with different colors representing different modules; (**C**): Heat map of the correlation between modules and traits; The values in each box represent the correlation coefficient R^2^ and *p*-value; Red indicates positive correlation, and blue indicates negative correlation. (**D**): Number of genes in each module.

WGCNA divided the genes into 12 co-expression modules (Figure 6A,B). Module–trait correlation analysis (Figure 6C,D) revealed that the antiquewhite4 module (3685 genes) was significantly negatively correlated with IAA, GA, and ABA contents, while the darkorange2 module (798 genes) was significantly positively correlated with CTK and GA contents. These two modules were identified as key modules regulating axillary bud germination.

### 2.6. Identification of Hub Transcription Factors in Target Modules

**Figure 7:**
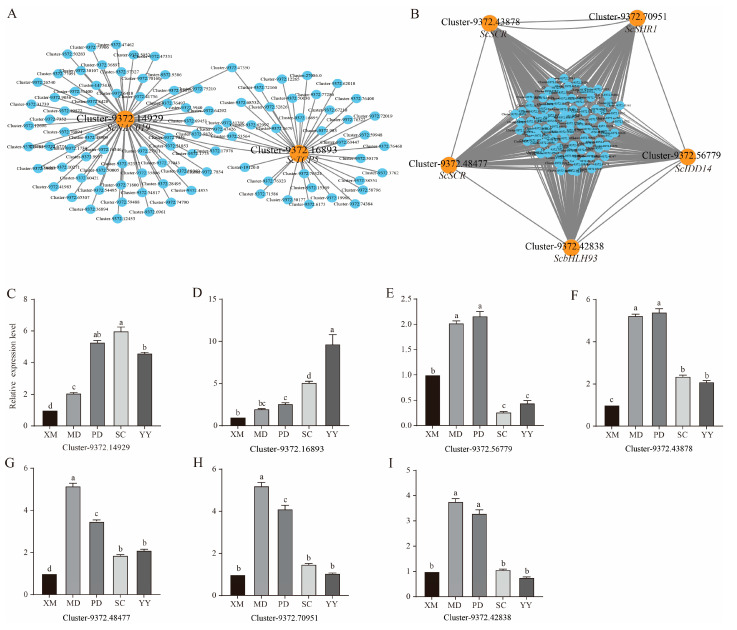
Gene co-expression networks of hub transcription factors and their expression patterns during axillary bud germination. (**A**) Co-expression network of the top 100 hub genes from the antiquewhite4 module. (**B**) Co-expression network of the top 100 hub genes from the darkorange2 module. In (**A**,**B**), nodes = genes, edges = co-expression relationships, and hub transcription factors are shown as larger orange nodes. (**C**–**I**) Expression levels of core transcription factors at five stages. Data are mean ± SEM (n = 3). Different letters above bars indicate *p* < 0.05 (one-way ANOVA). XM: dormancy period; MD: stirring period; PD: swelling period; SC: elongation period; YY: first new leaf period.

The top 100 hub genes with the highest kME values were selected from the antiquewhite4 and darkorange2 modules to construct co-expression networks (Figure 7A,B). Based on functional annotation and network connectivity, seven core transcription factors were ultimately identified (Table 2). In the antiquewhite4 module, the hub transcription factors *ScNAC019* (Cluster-9372.14929) and *ScTCP5* (Cluster-9372.16893) belonged to the NAC and TCP families, respectively. In the darkorange2 module, five hub transcription factors were identified, namely *ScbHLH93* (Cluster-9372.42838), *ScSCR* (Cluster-9372.43878, Cluster-9372.48477), and *ScSHR1* (Cluster-9372.70951). Notably, *ScSCR* and *ScSHR1* formed the canonical SHR–SCR regulatory module.

The expression patterns of the seven identified hub transcription factors at different stages of axillary bud germination and development were validated by RT-qPCR. The results showed that *ScNAC019* and *ScTCP5* were significantly upregulated from the swelling stage to the new leaf stage. *ScSHR1*, *ScSCR*, *ScIDD14*, and *ScbHLH93* were rapidly and significantly up-regulated during the swelling and sprouting stages, followed by a decline; however, their expression levels at the elongation and new leaf stages (except for *ScIDD14*) remained higher than those at the dormancy stage (Figure 7C–I).

### 2.7. RT-qPCR Validation of Transcriptome Data Reliability

**Figure 8:**
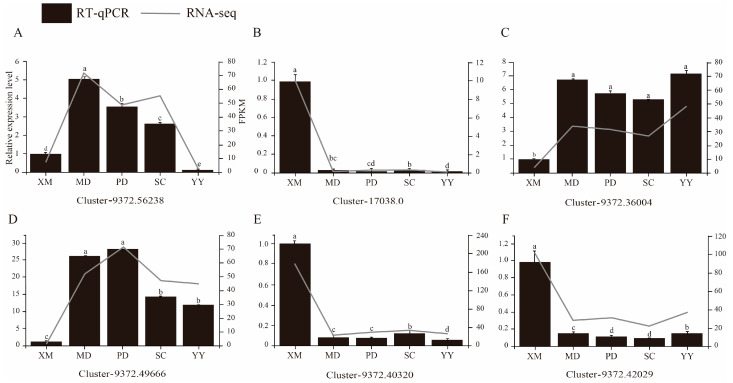
qRT-PCR and RNA-seq analysis of 6 DEGs. XM: dormancy period, MD: stirring period, PD: swelled period, SC: elongation period, YY: the first new leaf period. Statistical significance was determined by one-way ANOVA, and different letters above the bars indicate significant differences at *p* < 0.05.

To validate the reliability of the transcriptome data, six DEGs involved in the IAA, CTK, and ABA signaling pathways were selected for qRT-PCR analysis. Results showed (Figure 8) that *ScAUX1* (Cluster-9372.56238, IAA pathway) and *ScB-ARR* (Cluster-9372.49666, CTK pathway) exhibited upregulation followed by downregulation during axillary bud emergence. Conversely, *ScIAA9* (Cluster-17038.0, IAA pathway) and *ScSnRK2* (Cluster-9372.40320, ABA pathway), and *ScPYL* (Cluster-9372.42029, ABA pathway) were downregulated, while *ScA-ARR* (Cluster-9372.36004, CTK pathway) was upregulated. These findings are highly concordant with RNA-seq results, confirming the reliability of the transcriptome sequencing data.

## 3. Discussion

Phytohormones, as small-molecule regulators, participate in various physiological processes in plants, including growth and defense [24,25]. In this study, IAA and GA contents decreased continuously and significantly throughout the germination process, suggesting their dominant roles in maintaining bud dormancy. Conversely, the peak accumulation of CTK at the initial stage of germination, together with the decline in ABA content during the swelling stage, synergistically constituted a “germination initiation signal”. The subsequent increase in ETH content during the late stage of germination may be involved in organ differentiation. These results indicate that axillary bud germination is not determined by a single hormone but rather depends on the precise switching of antagonistic hormone ratios—such as IAA/CTK and GA/ABA—at specific developmental nodes.

The coordinated changes in auxin signaling components further support this model. GH3 family genes, which encode IAA-conjugating enzymes that inactivate free IAA [26], showed sustained downregulation throughout germination, indicating reduced conversion of active IAA to inactive conjugates. However, this feedback mechanism did not prevent the overall decline in IAA content, suggesting that reduced auxin biosynthesis or enhanced degradation is the primary cause. The expression of AUX/IAA genes was largely downregulated, particularly at later stages, which may relieve the transcriptional repression of ARF factors and promote bud outgrowth [27]. Collectively, these expression patterns are consistent with the established role of auxin as a negative regulator of axillary bud outgrowth [28,29].

Conversely, the dynamics of CTK content were tightly associated with the expression of its downstream signaling genes. Type-A ARR genes, which are primary cytokinin response regulators rapidly induced by cytokinin treatment in Arabidopsis [30], showed sustained upregulation during the early stages when CTK levels peaked (MD and PD) (Figure 5B). In rice, similar induction of OsRR genes occurs upon cytokinin application, and elevated cytokinin levels promote tiller bud outgrowth by counteracting auxin-mediated inhibition [31]. Importantly, the peak of CTK accumulation occurred earlier than the significant decline in IAA content, supporting the established antagonistic interaction between cytokinin and auxin in shoot branching regulation [28,32]. This temporal hierarchy suggests that early CTK accumulation serves as a key leading event that initiates downstream signaling and overcomes IAA-mediated inhibition. These findings provide direct theoretical targets for promoting early and vigorous growth in sugarcane through exogenous hormone regulation, such as foliar application of CTK or GA inhibitors at the seedling stage.

Transcriptome sequencing has been widely applied to elucidate the mechanisms underlying axillary bud development in rice [33,34], tobacco (*Nicotiana tabacum* L.) [35] and other plants [36,37]. This study reveals that sugarcane axillary bud emergence is accompanied by extensive gene expression reprogramming, with the number of differentially expressed genes (DEGs) increasing progressively during development, reflecting the complexity of the transition from dormancy to active growth. KEGG enrichment analysis identified hormone signaling, starch and sucrose metabolism, photosynthesis, and phenylpropanoid biosynthesis as core pathways consistently involved throughout development. This molecularly confirms that germination is an energy-consuming process requiring mobilization of stored energy and establishment of new metabolic flux. The sustained enrichment of starch and sucrose metabolism pathways indicates rapid hydrolysis of carbohydrates stored in embryonic tissues into soluble sugars, providing carbon skeletons and energy for cell division and elongation [38]. The upregulation of photosynthesis-antennary protein-related genes in the later stages of germination suggests that newly formed tissues are rapidly establishing photosynthetic autotrophic capabilities, achieving the critical transition from heterotrophy to autotrophy. The active phenylpropanoid biosynthesis pathway may be associated with cell wall lignification, enhanced stress resistance, and auxin transport regulation [39,40,41]. This study identified numerous key components in hormone signaling pathways, such as AUX/IAA, ARRs, DELLA, and PYL/PP2C. Their expression patterns were highly correlated with corresponding hormone content changes, forming a complete regulatory chain from hormone sensing to gene response. These findings indicate that axillary bud emergence represents the synergistic advancement of three core biological processes at the gene expression level: hormone signal redistribution, energy metabolism restart, and functional establishment of newly formed organs.

Through WGCNA, we successfully condensed complex gene expression data into co-expression modules with clear biological significance and identified core modules significantly associated with key hormone traits. The antiquewhite4 module, which was significantly negatively correlated with IAA, GA, and ABA, together with the darkorange2 module, which was significantly positively correlated with CTK, formed the core of the regulatory network. This finding confirms, from a systems biology perspective, that hormones are the primary drivers of co-expression network dynamics. Hub transcription factors further identified from the key modules—such as *ScTCP5*, *ScNAC019*, *ScSCR*, and *ScSHR1*—serve as crucial nodes connecting upstream hormone signals with downstream growth and developmental programs. The sustained high expression of *ScTCP5* and *ScNAC019* suggests that these two transcription factors are involved in the entire process of axillary bud germination and development, facilitating its successful progression. TCP transcription factors have been reported to integrate brassinosteroid signals to regulate cell proliferation in Arabidopsis [42].

In this study, the co-expression network of *ScTCP5* was enriched in photosynthesis- and cell cycle-related genes, suggesting its potential core role in coordinating the transition of sugarcane axillary buds from dormancy to photoautotrophic growth. More importantly, we identified the canonical SHR–SCR module, which is conserved and critical for root meristem maintenance and stem cell niche regulation [43,44]. Its high expression in sugarcane axillary buds strongly implies that it also plays a central role in regulating the activation and maintenance of lateral meristems (axillary buds). The discovery of these hub transcription factors effectively links discrete hormone signaling events with specific developmental programs governing cell division, differentiation, and organogenesis, thereby providing core components for constructing a multi-layered regulatory network model of axillary bud germination in sugarcane.

## 4. Materials and Methods

### 4.1. Plant Materials and Processing

The commercial sugarcane variety XTT22 was used in this study. Plants were grown under standard agronomic practices in the experimental fields of the Sugarcane Research Institute, Yunnan Academy of Agricultural Sciences, Kaiyuan, China (23°42′ N, 103°16′ E). Healthy, 320-day-old (mature stage), field-grown plants were selected for sampling. To induce axillary bud germination, stem segments (approximately 8 cm in length) containing a single intact axillary bud were excised from the middle to upper portions of 20 randomly selected mother plants to account for biological variation. After surface sterilization in 1% carbendazim solution (Hangzhou JiaBiQi Biotechnology Co., Ltd., Hangzhou, China) for 5 min, followed by three rinses with sterile distilled water, the segments were placed horizontally on trays lined with three layers of moist sterile gauze. Germination was induced in a constant-temperature incubator (MGC-350HP-2, Shanghai Hengke Scientific Instrument Co., Ltd., Shanghai, China) at 28 °C with 70% relative humidity in the dark. Axillary bud tissues were collected at five developmental stages defined by morphological changes: dormancy (XM, bud not swollen), initiation (MD, bud tip showing green coloration), swelling (PD, bud body visibly enlarged), elongation (SC, bud body elongated), and first new leaf stage (YY, new leaf visible at bud tip). At each stage, approximately 20 buds were pooled to form one biological replicate, and three independent biological replicates were collected. All samples were immediately frozen in liquid nitrogen and stored at −80 °C until use for transcriptome sequencing and hormone analysis.

### 4.2. Measurement of Endogenous Hormones

The contents of IAA, GA, ABA, and CTK were determined using high-performance liquid chromatography (HPLC) following the method described by Gao et al. [45]. HPLC conditions: analyses were performed on a Rigol L3000 HPLC system (Rigol Technologies, Beijing, China) equipped with a Kromasil C18 reversed-phase column (250 mm × 4.6 mm, 5 μm). The mobile phase consisted of methanol and 1% acetic acid aqueous solution (30:70, *v*/*v*) at a flow rate of 0.8 mL/min. The injection volume was 10 μL, the column temperature was maintained at 30 °C, and the detection wavelength was set at 254 nm, with a run time of 50 min. The column was equilibrated with the mobile phase until a stable baseline was achieved before sample injection. Hormone extraction: Frozen bud samples (approximately 0.4 g) were ground in a mortar and extracted overnight at 4 °C with 1 mL of pre-cooled methanol:water:acetic acid (80:20:1, *v*/*v*/*v*). The homogenate was centrifuged at 8000× *g* for 10 min. The residue was re-extracted with 0.5 mL of the same solvent for 2 h and centrifuged again. The supernatants were combined and evaporated to dryness under a nitrogen stream at 40 °C. The residue was decolorized three times with 0.5 mL of petroleum ether, and the upper ether phase was discarded. The lower aqueous phase was adjusted to pH 2.8 with saturated citric acid solution and extracted three times with equal volumes of ethyl acetate. The organic phase was collected, evaporated to dryness under nitrogen, and redissolved in 0.5 mL of methanol. The solution was filtered through a syringe filter into a sample vial with an insert for HPLC analysis. Ethylene (ETH) content was determined according to the method described by Wang et al. [46], using a Thermo Scientific Trace 1310 gas chromatograph (Thermo Fisher Scientific, Waltham, MA, USA). Three replicates were performed per sample. Analyses were performed on a gas chromatograph equipped with a hydrogen flame ionization detector. The injector temperature was set at 60 °C, the detector temperature at 150 °C, and the column oven temperature at 60 °C. After ignition and baseline stabilization for 30 min, samples were injected for analysis.

### 4.3. RNA Extraction, Library Preparation, and Sequencing

Total RNA was extracted using the TRIzol^®^ Kit (Coolaber, Beijing, China). mRNA was enriched using oligo(dT) magnetic beads, fragmented, and converted into cDNA with the PrimeScript™ II 1st Strand cDNA Synthesis Kit (TaKaRa, Dalian, China) to construct the library. Double-end sequencing was performed on the Illumina HiSeq^TM^ 2000 platform (Illumina, Inc., San Diego, CA, USA) with three biological replicates per sample.

### 4.4. Transcriptome Data Analysis

Perform de novo assembly of high-quality clean reads using Trinity software (v2.15.1) and obtain non-redundant unigenes through hierarchical clustering with Corset. Functional annotation was performed using the NR, NT, Swiss-Prot, KOG/COG, Pfam, GO, and KEGG databases. Differential expression analysis was conducted using the R package DESeq2 (v1.26.0), with dormancy (XM) as the control. Genes were selected based on the criteria |log_2_(Fold Change)| ≥ 2 and a corrected *p*-value (FDR) ≤ 0.001. TBtools-II (v2.332) [47] was employed for Gene Ontology (GO) functional enrichment analysis and Kyoto Encyclopedia of Genes and Genomes (KEGG) pathway enrichment analysis, as well as visualization of differentially expressed genes (DEGs). Pathways with *p* adjust < 0.05 were considered significantly enriched, and the top 20 significantly enriched pathways were displayed.

### 4.5. Weighted Gene Co-Expression Network Analysis (WGCNA)

Using the Fragments Per Kilobase of transcript per Million mapped reads (FPKM) values for all genes as input, the gene co-expression network was constructed using the WGCNA R package [48]. A soft threshold β = 20 was selected to build the adjacency matrix and the Topological Overlap Matrix (TOM). Co-expression modules were partitioned using dynamic tree cutting (minimum gene count = 30). The correlation between module feature vectors and endogenous hormone content was calculated to screen significantly correlated modules (|Correlation| > 0.6, *p* < 0.05). Hub genes with the highest connectivity (kME values) were extracted from key modules. Gene interaction networks were visualized using Cytoscape (3.9.1) [49].

### 4.6. Real-Time Quantitative PCR Validation

Six differentially expressed genes involved in the IAA, CTK, ABA, and GA signaling pathways were randomly selected (primer sequences are listed in Table 3). *GAPDH* was used as the housekeeping gene [50], and real-time quantitative PCR was performed on an ABI 7500 real-time PCR system (Applied Biosystems, Foster City, CA, USA) using the SYBR Green method. Reaction systems and procedures followed the kit instructions (Vazyme Biotech Co., Ltd., Nanjing, China). Each sample included three technical replicates and three biological replicates. Relative expression levels were calculated using the 2^–ΔΔCt^ method [51]. Additionally, qRT-PCR was employed to validate the expression patterns of key hub transcription factors identified by WGCNA across different axillary bud development stages (primer sequences are listed in Table 3).

### 4.7. Statistical Analysis

Hormone data and qRT-PCR data were analyzed using Microsoft Excel 2022 (Microsoft Corp., Redmond, WA, USA) and GraphPad Prism 9.5 (GraphPad Software, San Diego, CA, USA) and expressed as mean ± standard error of the mean (SEM). One-way analysis of variance (ANOVA) with Tukey’s post hoc test was performed using GraphPad Prism 9.5, and statistical significance was defined as *p* < 0.05. PCA analysis was employed using R 4.1.0 (R Foundation for Statistical Computing, Vienna, Austria).

## 5. Conclusions

This study provides a systematic characterization of the hormonal and transcriptional dynamics governing axillary bud germination in sugarcane. Our results reveal that axillary bud outgrowth is associated with a coordinated decline in IAA and GA levels, coupled with a transient accumulation of CTK at the initiation stage, suggesting that a hormonal switch involving antagonistic hormone ratios—particularly IAA/CTK and GA/ABA—is critical for dormancy release. Transcriptomic analysis further demonstrates that this hormonal shift triggers extensive reprogramming of gene expression, with differentially expressed genes enriched in pathways related to hormone signal transduction, starch and sucrose metabolism, and photosynthesis, reflecting the transition from heterotrophic dormancy to autotrophic growth. Through WGCNA, we identified two key co-expression modules (antiquewhite4 and darkorange2) that are significantly correlated with hormone dynamics. Seven hub transcription factors, including *ScTCP5*, *ScNAC019*, and the conserved *ScSCR*-*ScSHR1* regulatory module, were identified as central nodes linking hormone signaling to downstream developmental programs. Notably, the presence of the SHR-SCR module in axillary buds suggests a conserved mechanism for meristem maintenance across different organs. Collectively, these findings establish a foundation for understanding the molecular regulatory network underlying axillary bud germination in sugarcane and provide candidate gene resources for molecular breeding aimed at improving tillering and yield in this important crop.

## Figures and Tables

**Table 1:** Analysis results of sugarcane axillary bud transcriptome data.

**Sample**	**Raw Reads**	**Clean Reads**	**Clean Bases (G)**	**Q20 (%)**	**Q30 (%)**	**GC (%)**	**Total Mapped (%)**
XM_1	88,334,526	86,514,672	12.98	97.63	93.48	55.7	72.47
XM_2	74,355,154	70,290,218	10.54	97.38	92.92	54.33	71.03
XM_3	75,649,536	73,951,168	11.09	97.54	93.24	55.99	72.68
MD_1	79,617,404	78,099,824	11.71	97.54	93.22	54.92	72.86
MD_2	73,451,474	71,750,402	10.76	97.33	92.79	54.62	72.4
MD_3	78,058,388	76,425,258	11.46	97.58	93.33	54.7	73.43
PD_1	75,362,334	73,694,718	11.05	97.84	93.94	55.71	73.28
PD_2	77,882,300	75,579,492	11.34	97.58	93.31	54.32	73.18
PD_3	68,589,340	66,876,620	10.03	97.76	93.77	55.48	73.43
SC_1	87,458,682	85,783,054	12.87	97.82	93.92	56.29	73.85
SC_2	81,759,250	80,022,292	12.00	97.77	93.79	56.22	74.28
SC_3	70,731,274	68,792,736	10.32	97.97	94.24	56.13	74.39
YY_1	76,666,988	74,798,808	11.22	97.74	93.7	54.92	72.36
YY_2	74,456,704	72,587,290	10.89	97.47	93.07	54.95	72.63
YY_3	68,965,284	67,630,526	10.14	97.48	93.08	54.82	72.5

**Table 2:** Functional annotation of hub transcription factors in the target module.

**Module**	**Gene ID**	**Gene Name**	**Gene Function**	**Family**
antiquewhite4	Cluster-9372.14929	*NAC019*	NAC transcription factor family	NAC
	Cluster-9372.16893	*TCP5*	Participates in the heterochronic regulation of leaf development and controls the morphogenesis of leaf margins.	TCP
darkorange2	Cluster-9372.48477	*SCR*	Regulating meristem fate	GRAS
	Cluster-9372.43878	*SCR*	Regulating meristem fate	GRAS
	Cluster-9372.70951	*SHR1*	Stem cell niche maintenance and cell fate determination are regulated by core transcription factors, forming an SHR-SCR regulatory module with SCR.	GRAS
	Cluster-9372.42838	*bHLH93*	bHLH_SF superfamily	bHLH
	Cluster-9372.56779	*IDD14*	Regulates starch metabolism, lateral organ morphogenesis, geotropism, and participates in establishing auxin gradients.	C2H2

**Table 3:** Primers used for qRT-PCR analysis.

**Gene ID**	**Primer Name**	**Primers (5′→3′)**
Cluster-9372.16893	ScTCP5-F	GGCGGCTAGTAAGACGATGATAA
Cluster-9372.16893 Cluster-9372.14929	ScTCP5-R	CTTGCTAGGCTGGTTGAGGC
	ScNAC019-F	AACGATGTCAACAGCGGAAGC
Cluster-9372.14929 Cluster-9372.43878	ScNAC019-R	TCGTCAGTATTGGGAGGAGGTG
	ScSCR-F	CGAGCTTGTCCGGGTCAACA
Cluster-9372.43878 Cluster-9372.48477	ScSCR-R	TGGCCGGGACTCTTCCACAT
	ScSCR-F	GAGGGGAACATTCCCAGCAG
Cluster-9372.48477 Cluster-9372.70951	ScSCR-R	CGTGGAGGCCATCCACTACTA
	ScSHR-F	GCAGGACACGAGGTCCACAA
Cluster-9372.70951 Cluster-9372.56779	ScSHR-R	AAGCAGCCAACACGGAGACG
	ScIDD14-F	AGCTTCATAGAGCACCAGGACAC
Cluster-9372.56779 Cluster-9372.42838	ScIDD14-R	GCGGTCGTAGGGCTTGGAAT
	ScbHLH93-F	CGGCGTCCAAGAAGAAGAGG
Cluster-9372.42838 Cluster-9372.36004	ScbHLH93-R	AGCTCCCGGAAGACGCTAAG
	ScA-ARR-F	TGACGGTGGTGGATGCC
Cluster-9372.36004 Cluster-9372.49666	ScA-ARR-R	TCACTTGGTAGGACGAGTTCTTG
	ScB-ARR-F	ATCCTGTCAGTTGTGATCTGTTCCC
Cluster-9372.49666 Cluster-9372.40320	ScB-ARR-R	GAGATGACTGGCTACGACCTGC
	ScSnRK2-F	CTTGTTGGTGCTTATCCCTT
Cluster-9372.40320 Cluster-17038.0	ScSnRK2-R	TCTGGTACTCATCGGTCATCT
	ScIAA9-F	GCCAAGTACGTGAAGGTGAAGAA
Cluster-17038.0 Cluster-9372.56238	ScIAA9-R	TCCGACCAGCATCCAGTCCC
	ScAUX1-F	AGGGAGAACGCCGTGGAGC
Cluster-9372.56238 Cluster-9372.42029	ScAUX1-R	GCACTGGTAGCACCTGGTGAAGA
	ScPYL-F	AGGAAGGCCGAGATGGTGG
Cluster-9372.42029 *GAPDH*	ScPYL-R	TTGATGTGCTTGACGAGGGTG
	GAPDH-F	CACGGCCACTGGAAGCA
*GAPDH*	GAPDH-R	TCCTCAGGGTTCCTGATGCC

## Data Availability

Raw data are available upon request.
